# Effects of Novel Anti-VEGF Agents with Intravitreal Conbercept in Diabetic Retinopathy: A Systematic Review and Meta-Analysis

**DOI:** 10.1155/2021/9357108

**Published:** 2021-02-03

**Authors:** Hui Wang, Jing Zhou, Caoyu Sun, Xu Dong

**Affiliations:** Department of Ophthalmology, The 4th People's Hospital of Shenyang, Shenyang, Liaoning 110031, China

## Abstract

To evaluate the efficacy and safety of intravitreal conbercept (IVC) for diabetic retinopathy (DR) compared with intravitreal triamcinolone acetonide (IVTA). PubMed, Embase, Cochrane Library, China National Knowledge Infrastructure, VIP database, and Wanfang database were searched from their earliest records to January 2020. We included randomized controlled trials (RCTs) evaluating the efficacy and safety of conbercept in DR patients compared with ITVA. Outcomes included the mean changes from the baseline in best corrected visual acuity (BCVA) score, central macular thickness (CMT), quality of life (QoL) over time, and the incidence of adverse events (AEs). A total of 19 RCTs involving 1,811 eyes were included in this meta-analysis. IVC might improve BCVA (WMD = 0.10, 95% CI (0.07, 0.12), *P* < 0.001) and reduce CMT (WMD = −102.5, 95% CI (−148.48, −56.53), *P* < 0.001) compared to IVTA. The incidence of AEs in patients receiving IVC was significantly lower than those receiving IVTA (RR = 0.29, 95% CI (0.21, 0.40), *P* < 0.001). Patients with IVC treatments acquired better self-care, mobility, social, and mental scores compared with IVTA (*P* < 0.001). Current evidence shows that IVC has better effects and safety than IVTA in treating DR, and it can significantly enhance the QoL of patients with DR.

## 1. Introduction

Diabetic retinopathy (DR) is one of the most common microvascular complications of diabetes and will result in visual impairments for patients with diabetes. It has become the leading cause of blindness in working-age adults in developed countries [1, 2]. The incidence of DR is expected to be 642 million by 2040, highlighting the overwhelming burden to society [3]. A prevalence meta-analysis revealed that the global prevalence of DR, nonproliferative DR (NPDR), and proliferative DR (PDR) was 28%, 27%, and 6% in people with type 2 diabetes (T2D), respectively [4]. These estimates are expected to rise further due to the increasing prevalence of diabetes and life expectancy of those with diabetes.

Previously, alternatives for eyes that have a suboptimal response to vascular endothelial growth factor (VEGF) inhibitors include corticosteroids such as triamcinolone acetonide (TA). Intravitreal triamcinolone acetonide (IVTA) was the widely used medication for diabetic macular edema (DME) [5]. Recently, newly established therapies for PDR and DR related macular edema is intravitreous injection of a vascular endothelial growth factor (VEGF) agency. According to the reports from American Academy of Ophthalmology (AAO) and the European Society of Retina Specialists (EURETINA), VEGF inhibitors are most effective to manage DME [6, 7]. VEGF plays a critical role in neovascularization and enhancing the inflammatory response. Inhibiting VEGF can limit the angiogenesis of DR [8]. Conbercept, produced by Chengdu Kang Hong Biotech (Sichuan, China), is a recombinant fusion protein with high affinity to both VEGF-A and VEGF-B isoforms and PIGF [9]; it was developed and approved by the China Food and Drug Administration (CFDA) for the treatment of DME in 2019. Several studies concluded that IVC was effective and safe in the treatment of DR and DME. However, current evidence has not been systematically assessed; thus, a systematic review and meta-analysis was conducted to evaluate the efficacy and safety of IVC in treatment of DR.

## 2. Methods

### 2.1. Search Strategy

This meta-analysis followed the standard set of Preferred Reporting Items for Systematic reviews and Meta-Analyses (PRISMA).

### 2.2. Literature Searching

PubMed, Cochrane Library, Embase, China Knowledge Network (CNKI), Wanfang, and VIP database were independently searched by two researchers (HW and JZ) from their earliest records to February 2020. “Conbercept” and “triamcinolone acetonide” AND “diabetic retinopathy” or “diabetic macular edema” were used as search terms.

Inclusion criteria included randomized controlled trial (RCT) of conbercept vs. triamcinolone in the treatment of DR; publication written in English or Chinese; outcome indicators which are the best corrected visual acuity, central macular thickness, the incidence of adverse events (AEs), and quality of life score.

Exclusion criteria were listed as follows: retrospective study, nonrandomized controlled study, studies without available raw data, duplicated literature, and review, comment, animal experiment research, and conference abstract.

### 2.3. Data Extraction and Quality Evaluation

Two researchers (HW and JZ) independently reviewed the titles and abstracts of the eligible articles identified by literature searching and assessed the studies. The full texts of relevant articles were retrieved for detailed screening. Any disagreement was resolved by discussion. Two investigators independently extracted data from included articles as follows: (1) first authors, (2) year of publication, (3) country or location, (4) study design, (5) number of participants in each group, (6) gender and age of participants; (7) treatment regimens, and (8) outcomes of each study. The modified Jadad scale was used to evaluate the quality of the included studies. The specific methods are as follows: the randomization method properly assigns 2 points, unclearly assigns 1 point, and inappropriately assigns 0 points; binding assignment concealment properly assigns 2 points, unclearly assigns 1 point, and inappropriately assigns 0 points; appropriately assigns 2 points, unclearly assigns 1 point, and improperly assigns 0 points; and withdraw and exit describe assigning 1 point and do not describe assigning 0 points. The scores range from 1 to 7, with 1–3 for low-quality studies and 4–7 for high-quality studies.

### 2.4. Statistical Analysis

STATA (v.11.0; StataCorp LP, College Station, TX) was used for quantitative comprehensive analysis of the included research literature. Categorical variables use hazard ratio (RR) as the effect size, while continuous variables use weighted mean difference (WMD), and each effect size is expressed within 95% confidence interval (CI). Statistical heterogeneity among studies was examined by the chi-square test and quantified by the *I*^2^ statistic. When *P* ≥ 0.1 and *I*^2^ ≤50%, there was no statistical heterogeneity between studies. We used the random effect model for meta-analysis. *P* < 0.05 indicates that the difference is statistically significant. Begg's test analysis was used to evaluate publication bias.

## 3. Results

### 3.1. Characteristics of Included Studies

**Figure 1:**
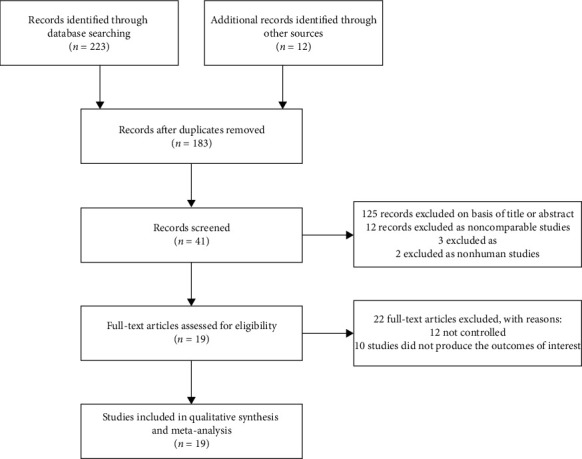
Flow diagram of included studies for this meta-analysis.

A total of 235 articles were initially obtained, and 183 articles were left after excluding duplicate ones. After screening the title and abstract, 41 articles were left. After reading the full text, 19 articles [10–28] involving 1,811 eyes were finally included according to the predetermined inclusion criteria (Figure 1). The characteristics of included studies are shown in Table 1. All the RCTs were single-center studies conducted in China. The follow-up time ranged from 1 to 6 months after the first treatment. The reported dose of conbercept ranged from 0.5 to 1.0 mg.

The 19 RCTs included in this study reported the patient's baseline status and were comparable. 12 studies mentioned randomness in the text, and the rest of the literature did not describe the specific random methods. All studies did not explain whether the allocation concealment was hidden. The specific quality evaluation results are shown in Table 2.

### 3.2. Best Corrected Visual Acuity (BCVA)

**Figure 2:**
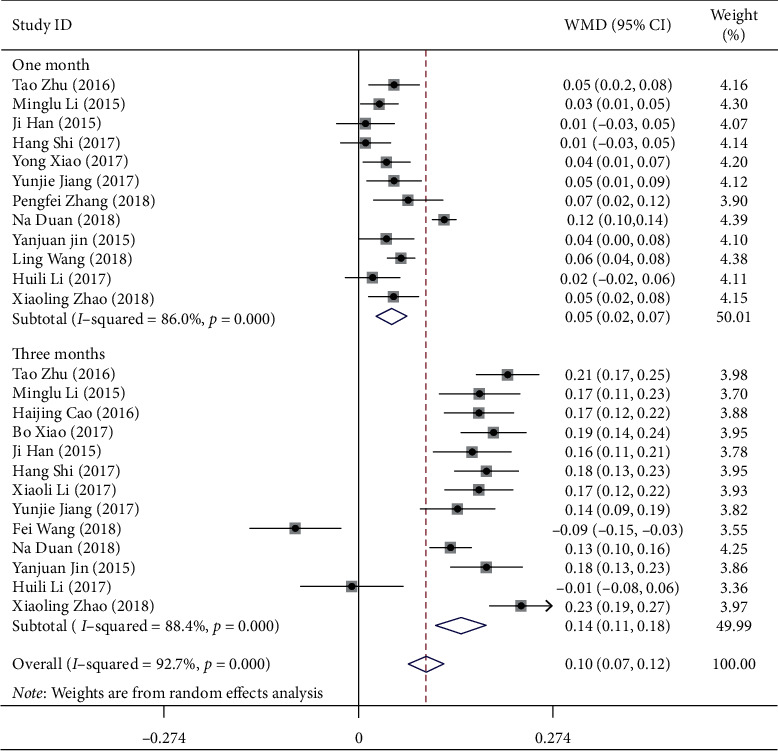
Forest plot showing the mean change in BCVA from baseline to one month after the first treatment.

The mean change in BCVA from baseline to the one month after the first treatment was reported in 12 studies, while the third month outcomes was reported in 13 studies (Figure 2). The difference between the pooled results of the two subgroups (one month and three months) was significant (*P* < 0.001). Compared to IVTA, IVC significantly improved the BCVA in both the first (WMD = 0.11, 95% CI (0.02, 0.07)) and third month after the treatment (WMD = 0.14, 95% CI (0.11, 0.18)).

### 3.3. Central Macular Thickness (CMT)

**Figure 3:**
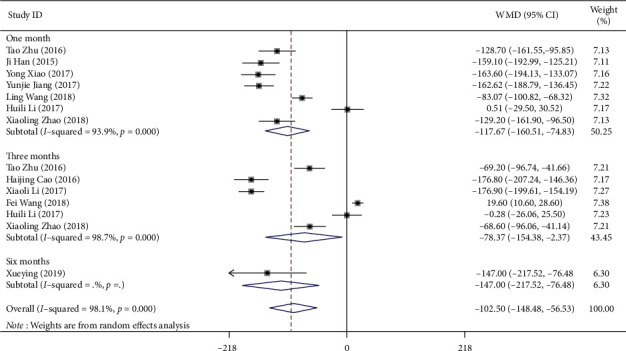
Forest plot showing the subgroup analyses on central macular thickness stratified by follow-up selection.

There were seven, six, and one studies that reported the mean change in CMT from baseline to the last visit at one, three, and six months, respectively. Subgroup analyses were performed and stratified by follow-up selection (Figure 3). There were significant differences among the pooled results of the three subgroups (*P* < 0.001). Compared with IVTA therapy, IVC significantly reduced the CMT at the last visit (WMD = −117.67, 95% CI (−160.51, −74.83); WMD = −78.37, 95% CI (−154.38, −2.37); WMD = −147.00, 95% CI (−217.52, −76.48), respectively).

### 3.4. Quality of Life (QoL) Scores

**Figure 4:**
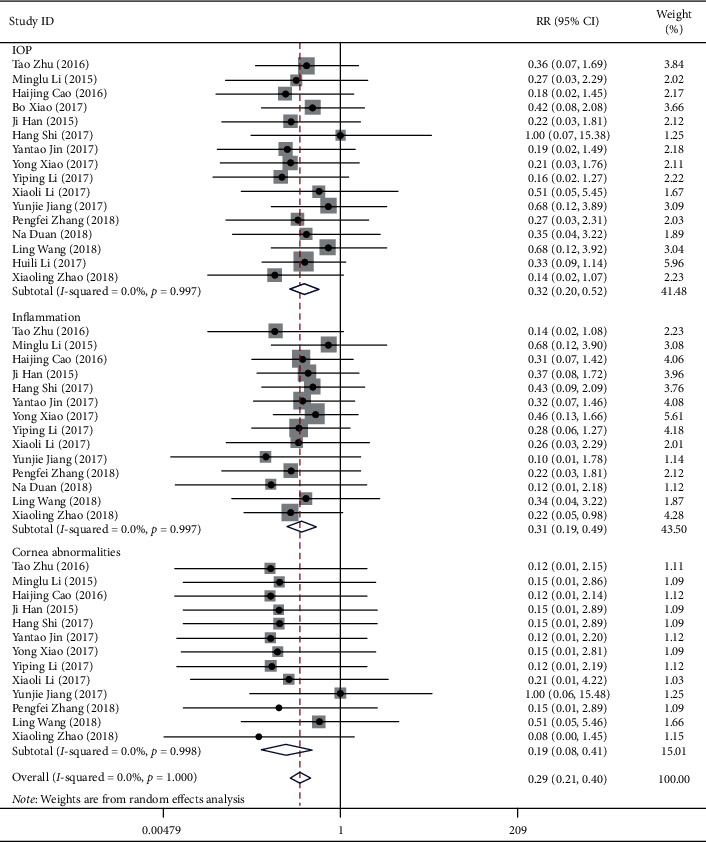
Forest plot showing the quality of life questionnaires after treatments.

There were eight studies that reported the QoL of participants after IVC or IVTA treatments (Figure 4). The QOL questionnaires divided into the following subscales: self-care, mobility, social, and mental. Patients with IVC treatments acquired better self-care (WMD = 8.95, 95% CI (4.59, 13.30)), mobility (WMD = 11.64, 95% CI (7.45, 15.84)), social (WMD = 9.821, 95% CI (6.65, 12.99)), and mental (WMD = 15.02, 95% CI (9.24, 20.80)) scores compared with IVTA at the last visit, respectively.

### 3.5. Adverse Events (AEs)

**Figure 5:**
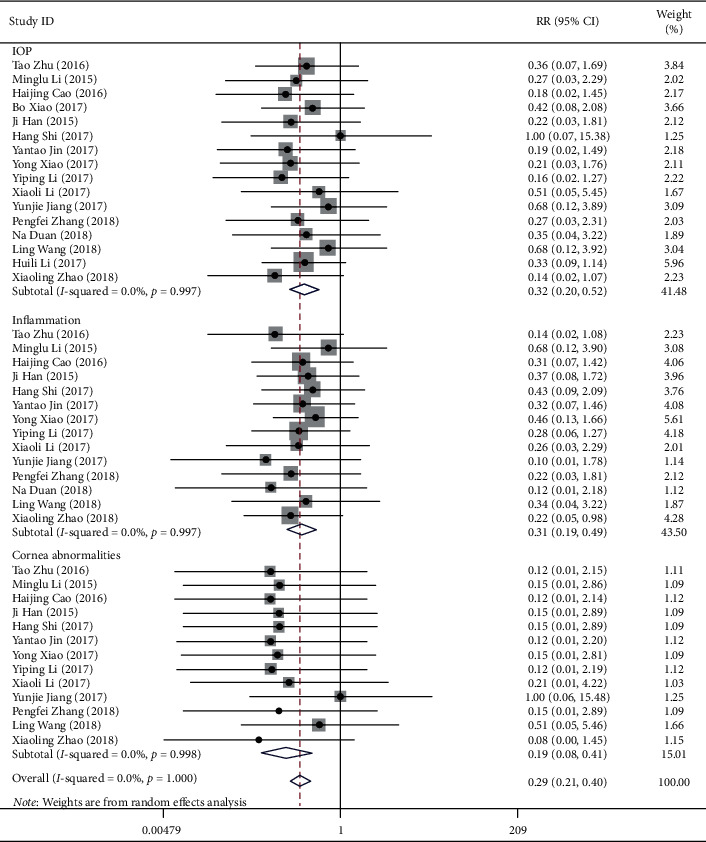
Forest plot showing the effect of the anti-VEGF method for diabetic retinopathy in adverse events.

17 studies reported the incidence of any AEs (Figure 5). The incidences of increased IOP, inflammation reaction, and cornea abnormalities in the IVC group were less than that in the IVTA group (RR = 0.32, 95% CI (0.20, 0.52); RR = 0.31, 95% CI (0.19, 0.49); RR = 0.19, 95% CI (0.08, 0.41), respectively).

### 3.6. Heterogeneity and Publication Bias

In this meta-analysis, there were significant heterogeneity detected except AEDs analysis (heterogeneity: *P* < 0.001; *I*^2^ >50%). To find the source of heterogeneity, the included studies were excluded one by one, and we found no significant changes in the heterogeneity or the results. In addition, Begg's and Egger's tests of the included studies suggested no significant publication bias in this meta-analysis (Table 3).

## 4. Discussion

In current systematic review and meta-analysis, by pooling the evidence of the available RCTs, we found that IVC significantly improved the BCVA, CMT, as well as the QoL score and decreased AEDs results of the patients with DR compared with IVTA therapy. Previous studies indicated that IVC therapy was associated with increase improvements of visual function, and the results of our study demonstrated that IVC therapy improved QoL scores, indicating that it may be an important measurement underlying the therapeutic effect of anti-VEGF agents.

Conbercept is a new anti-VEGF drug approved for the treatment of diabetic macular edema in China. Although it has been widely used in clinic, unlike other anti-VEGF drugs, validation and consensus on its clinical effects and safety have not been systematically sorted out. Triamcinolone is a corticosteroid hormone that is artificially synthesized and widely used in the treatment of macular edema and uveitis [29]. Central macular function in patients with acute DME improved after intravitreal triamcinolone injection [30]. In our meta-analysis, the measures of the variables in patients with DR receiving conbercept were superior to those receiving IVTA throughout the 1–6-month observation period. The findings of current meta-analysis indicate that after three months of treatment with IVC, appreciable BCVA improvement was achieved. Compared with IVTA, IVC therapy can significantly reduce the thickness of CMT. Thus, we speculate that IVC may have a better long-term effect in the treatment of DR than IVTA.

Intravitreal injection of conbercept can directly contact the retinopathy, increase the intraocular drug concentration, and significantly improve the treatment effect. Vision loss seriously affects the quality of life of patients. Interestingly, our study evaluated the QoL in patients after both IVC and IVTA treatment. It was found that the self-care ability, activity ability, and social and psychological scores of patients in the conbercept group were higher than those in the triamcinolone group, indicating IVC improves the patient's QoL.

Significant heterogeneity was present for many outcomes, which may be explained by differences in dosing methods or sample size. Studies that used a 1.0 ml IVTA injection, where typically the percentage of the higher levels of CMT, were found to show a much larger effect size of IVTA for this outcome, with more heterogeneity compared with studies using a 0.5 ml approach, where typically smaller starting doses are used. Given that a different dose would be anticipated to lead to higher and more widely varied effects of treatments, this may explain why heterogeneity was significant to some subgroups. Although sensitivity analysis showed no significant changes in the heterogeneity or the results, our findings should be noticed with cautious.

The strengths of this study include a comprehensive meta-analysis detecting the effect of IVC in the treatment of DR with both visual function and quality of life indicators, encompassing a total of 19 RCTs, and no publication bias was detected by Begg's or Egger's tests. However, there were some limitations in current meta-analysis. First, all included studies were from China. Second, the longest observation of the included studies was 6 months. Considering that DR is a chronic disease, longer follow-up is required. Third, the data were insufficient to conduct a dose-response meta-analysis. Furthermore, additional well-designed studies are still needed in the future.

## 5. Conclusion

Based on the findings from our meta-analysis, IVC therapy significantly improves BCVA and QoL scores, while it decreases CMT and AEDs in treatments for DR compared with IVTA therapy. Consequently, our results suggest that IVC is effective and safe in treatment of DR. Accordingly, IVC might be a potential therapy for patients with DR to improve QoL. Longer, well-designed, multicenter RCTs are needed to confirm our findings.

## Figures and Tables

**Table 1:** Study characteristics of included studies.

Author (year)	Study design	No. of participants (male/female)		No. of eyes		Age (years)		Dosage of drug		Follow-up (months)	Baseline BCVA of case		Baseline BCVA of control		Baseline CMT of case		Baseline CMT of control	
		IVC	IVTA	IVC	IVTA	IVC	IVTA	IVC	IVTA		Mean	SD	Mean	SD	Mean	SD	Mean	SD
Tao Zhu (2016) [10]	Prospective	32/27	31/28	59	59	42.3 ± 7.2	43.1 ± 6.8	0.1 ml	0.1 ml	3	0.07	0.02	0.07	0.01	643.1	105.9	638.9	102.8
Minglu Li (2015) [11]	Prospective	27/20	26/21	47	47	47.9 ± 2.8	47.3 ± 2.2	0.05 ml	0.1 ml	3	0.06	0.03	0.06	0.01	N.a	N.a	N.a	N.a
Haijing Cao (2016) [12]	Prospective	23/21	24/19	44	43	46.2 ± 6.3	45.5 ± 5.8	0.1 ml	0.1 ml	3	0.06	0.02	0.06	0.01	803.6	129.7	804.2	132.5
Bo Xiao (2017) [13]	Prospective	25/31	27/29	56	56	58.5 ± 4.2	59.1 ± 3.1	0.05 ml	0.05 ml	3	N.a	N.a	N.a	N.a	N.a	N.a	N.a	N.a
Ji Han (2015) [14]	Prospective	20/16	19/17	36	36	46 ± 2.2	45.6 ± 2.3	0.1 ml	0.1 ml	3	0.06	0.01	0.06	0.02	803.4	129.4	804.9	133.8
Hang Shi (2017) [15]	Prospective	20/15	21/14	35	35	45.8 ± 12.1	46.2 ± 12.2	0.1 ml	0.1 ml	6	0.06	0.01	0.07	0.02	N.a	N.a	N.a	N.a
Yantao Jin (2017) [16]	Prospective	21/19	22/18	40	40	65.2 ± 10.3	65.4 ± 10.5	0.1 ml	0.1 ml	N.a	0.06	0.03	0.06	0.02	805.2	132.8	805.4	133
Yong Xiao (2017) [17]	Prospective	27/17	24/19	44	43	50.1 ± 4.9	51.1 ± 5.2	0.1 ml	0.1 ml	3	0.05	0.02	0.06	0.03	806.2	126.6	806.3	132.2
Yiping Li (2017) [18]	Prospective	24/19	26/17	43	43	12.8 ± 4.9	61.3 ± 15.5	0.05 ml	0.1 ml	3	N.a	N.a	N.a	N.a	N.a	N.a	N.a	N.a
Xiaoli Li (2017) [19]	Prospective	30/22	30/22	52	52	50.3 ± 3.2	50.5 ± 3.3	0.1 ml	0.1 ml	3	0.05	0.02	0.05	0.03	804.2	123.5	803.5	125.6
Yunjie Jiang (2017) [20]	Prospective	22/20	24/18	42	42	51.6 ± 11.2	50.8 ± 10.6			3	0.06	0.03	0.06	0.02	803.5	125.7	804.6	128.6
Pengfei Zhang (2018) [21]	Prospective	N.a	N.a	37	37	49.20 ± 3.32	49.12 ± 3.12	0.1 ml	0.1 ml	1	0.07	0.02	0.08	0.01	188.8	40.1	187.8	41.2
Fei Wang (2018) [22]	Prospective	11/8	10/9	19	19	56.4 ± 11.2	55.8 ± 10.1	0.1 ml	0.1 ml	3	N.a	N.a	N.a	N.a	N.a	N.a	N.a	N.a
Na Duan (2018) [23]	Prospective	29/19	32/16	48	48	61.90 ± 8.23	61.76 ± 8.15	0.1 ml	0.1 ml	3	0.06	0.01	0.06	0.02	N.a	N.a	N.a	N.a
Yanjuan Jin (2015) [24]	Prospective	26/23	21/28	49	49	48.3 ± 6.4	47.8 ± 6.9	0.05 ml	0.05 ml	3	0.06	0.01	0.06	0.02	N.a	N.a	N.a	N.a
Linig Wang (2018) [25]	Prospective	31/29	40/20	60	60	65.09 ± 8.12	64.02 ± 9.16	0.05 ml	0.1 ml	1	0.07	0.02	0.08	0.03	692.91	98.23	699.37	97.81
Xueying Ji (2019) [26]	Prospective	33/27	32/28	60	60	45.39 ± 4.22	45.87 ± 5.19	0.05 ml	0.05 ml	6	N.a	N.a	N.a	N.a	514.11	247.43	513.87	218.48
Huili Li (2017) [27]	Prospective	40/36	42/34	76	76	54.43 ± 6.48	54.24 ± 6.33	0.05 ml	0.1 ml	3	0.13	0.09	0.14	0.1	586.42	134.21	587.19	136.32
Xiaoling Zhao (2018) [28]	Prospective	32/28	31/28	60	59	42.6 ± 7.3	43.2 ± 6.7	0.05 ml	0.1 ml	3	0.06	0.01	0.06	0.02	643.5	105.7	638.7	102.6

IVC, intravitreal conbercept; IVTA, intravitreal triamcinolone acetonide; SD, standard deviation; N.a, not applicable; BCVA, best corrected visual acuity; CMT, central macular thickness.

**Table 2:** The study quality of the included studies assessed by the JADAD score.

Author	Randomized	Allocation concealment	Blindness	Withdraw	JADAD score
Tao Zhu (2016) [10]	Yes	N.A.	No	N.A.	4
Minglu Li (2015) [11]	Yes	N.A.	No	N.A.	4
Haijing Cao (2016) [12]	Yes	N.A.	N.A.	N.A.	5
Bo Xiao (2017) [13]	Yes	N.A.	No	N.A.	4
Ji Han (2015) [14]	Yes	N.A.	No	N.A.	4
Hang Shi (2017) [15]	N.A.	N.A.	No	N.A.	3
Yantao Jin (2017) [16]	N.A.	N.A.	No	N.A.	3
Yong Xiao (2017) [17]	N.A.	N.A.	No	N.A.	3
Yiping Li (2017) [18]	N.A.	N.A.	No	N.A.	3
Xiaoli Li (2017) [19]	Yes	N.A.	No	N.A.	4
Yunjie Jiang (2017) [20]	Yes	N.A.	No	N.A.	4
Pengfei Zhang (2018) [21]	N.A.	N.A.	No	N.A.	3
Fei Wang (2018) [22]	Yes	N.A.	No	N.A.	4
Na Duan (2018) [23]	Yes	N.A.	No	N.A.	4
Yanjuan Jin (2015) [24]	N.A.	N.A.	No	N.A.	3
Linig Wang (2018) [25]	Yes	N.A.	No	N.A.	4
Xueying Ji (2019) [26]	Yes	N.A.	No	N.A.	4
Huili Li (2017) [27]	N.A.	N.A.	No	N.A.	3
Xiaoling Zhao (2018) [28]	Yes	N.A.	No	N.A.	4

**Table 3:** Potential publication bias by Egger's and Begg's tests.

Variables	Begg's	*P*	Egger's	*P*
BCVA				
One month	1.23	0.22	10.17	0.07
Three months	−0.24	0.81	4.27	0.47
Overall	0.64	0.52	7.61	0.05

CMT				
One month	−0.15	0.88	−5.28	0.46
Overall	−0.60	0.54	1.70	0.12

Complication				
IOP	0.90	0.37	0.17	0.79
Inflammation	−1.15	0.25	−0.65	0.06
Cornea abnormalities	16.39	0.37	−3.91	0.05
Overall	0.15	0.88	−0.57	0.05

Quality of life				
Social	0.25	0.81	4.77	<0.001
Mental	0.00	0.99	10.51	0.001
Self-care	−0.74	0.45	7.80	0.014
Mobility	1.24	0.22	14.18	<0.001
Overall	0.37	0.71	5.90	<0.001

BCVA, best corrected visual acuity; CMT, central macular thickness; IOP, intraocular pressure.

## Data Availability

The datasets generated and/or analyzed during the current study are available from the corresponding author upon request.
